# A cost‐utility analysis of avelumab for metastatic Merkel cell carcinoma in Taiwan

**DOI:** 10.1002/cnr2.1399

**Published:** 2021-05-02

**Authors:** Wen‐Cheng Chang, Amy Y. Lin, Jason C. Hsu, Chiao‐En Wu, Connie Goh, Patrick Chou, Kaitlin Kuo, Anne Chang, Roberto Palencia

**Affiliations:** ^1^ Division of Haematology‐Oncology, Department of Internal Medicine, Chang Gung Memorial Hospital at Linkou Chang Gung University College of Medicine Taoyuan Taiwan; ^2^ College of Medicine Chang Gung University Taoyuan Taiwan; ^3^ Merck Ltd. Taipei Taiwan; ^4^ Merck KGaA Darmstadt Germany; ^5^ International Ph.D. Program in Biotech and Healthcare Management, College of Management Taipei Medical University Taipei Taiwan; ^6^ IQVIA Solutions Taiwan Ltd. Taipei Taiwan

**Keywords:** avelumab, economic model, health technology assessment, JAVELIN Merkel 200 study, metastatic Merkel cell carcinoma, Taiwan

## Abstract

**Background:**

Metastatic Merkel cell carcinoma (mMCC) has traditionally been managed with palliative chemotherapy regimens or best supportive care (BSC). Avelumab, a novel anti‐programmed death‐ligand 1 (PD‐L1) human monoclonal antibody for mMCC treatment, is being studied in the pivotal JAVELIN Merkel 200 trial.

**Aim:**

Incorporating trial results, this analysis aimed to evaluate the cost‐utility of avelumab in Taiwan.

**Methods and results:**

A de novo partitioned‐survival model with three key health states related to survival (progression‐free disease, progressed disease, and death) was applied in this study. The data of clinical efficacy, safety, and patient utilities were obtained from the JAVELIN Merkel 200 trial, literature review, and Taiwanese clinical expert opinion. Cost‐utility analysis was performed, and results were presented as cost per quality‐adjusted life year (QALY) gained. For treatment‐naïve patients, the incremental cost‐effectiveness ratios (ICERs) for avelumab vs BSC and avelumab vs chemotherapy were US$44885.06 and US$42993.06 per QALY gained, respectively. As to treatment‐experienced mMCC patients, avelumab was associated with ICERs of US$27243.06 (vs BSC)/US$26557.43 (vs chemotherapy) per QALY gained. All ICERs remained consistently within the willingness‐to‐pay (WTP) threshold of US$53,333.33 per QALY gained.

**Conclusion:**

This study demonstrated avelumab to be a cost‐effective treatment option for both treatment‐experienced and treatment‐naïve mMCC patients with very poor prognosis in Taiwan.

## 1 INTRODUCTION

Merkel cell carcinoma (MCC) is an aggressive and ultra‐rare skin neuroendocrine carcinoma, and is associated with Merkel cell polyomavirus (MCPyV) infection, immunosuppression, and ultraviolet (UV) exposure.^1^, ^2^ The reports of MCC have been limited, particularly in Asians.^3^, ^4^, ^5^, ^6^ The accurate incidence or prevalence of MCC in Taiwan is unknown. Between 2000 and 2019, there were 24 MCC cases diagnosed and treated at the Chang Gung Memorial Hospital (CGMH).^6^ As this medical center covers about 34% of inpatients and 20% of outpatients with cancers in Taiwan,^7^, ^8^ the annual incidence of MCC could be estimated to be around 10 cases per year (4 per 10 000 000 persons). With limited therapeutic options available, patients with metastatic MCC (mMCC) are typically treated with palliative chemotherapy^9^, ^10^ or best supportive care (BSC). However, the median overall survivals (OSs) for chemotherapy‐naïve and chemotherapy‐experienced patients with mMCC were less than 12 and 6 months, respectively.^10^

Avelumab, a novel anti‐programmed death‐ligand 1 (PD‐L1) human monoclonal antibody, is being studied in the pivotal JAVELIN Merkel 200 trial in both chemotherapy‐refractory^11^ and chemotherapy naïve^12^ mMCC patients. Avelumab showed a favorable efficacy/safety profile with durable response in mMCC patients, and the objective response rates (ORRs) in 88 chemotherapy‐refractory patients^11^, ^13^ and 116 chemotherapy‐naïve patients^12^ were 33% and 39.7%, respectively. Compared with conventional chemotherapy, avelumab was associated with higher treatment response, longer survival, and more durable antitumor activity.^12^ Based on the JAVELIN Merkel 200 trial, avelumab has received approval for the treatment of mMCC in more than 40 countries worldwide, including Taiwan Food and Drug Administration (TFDA),^14^ US FDA,^15^ and European Medicines Agency (EMA) .^16^

This analysis aimed to evaluate the cost‐utility of avelumab upon national reimbursement in Taiwan. It described the steps that were followed to adapt the model in Taiwan, and presented the results of the adaptation of a partitioned‐survival economic model for avelumab compared with conventional care regimens for treatment‐naïve and treatment‐experienced mMCC patients.

## 2 METHODS

### 2.1 Model overview

**FIGURE 1:**
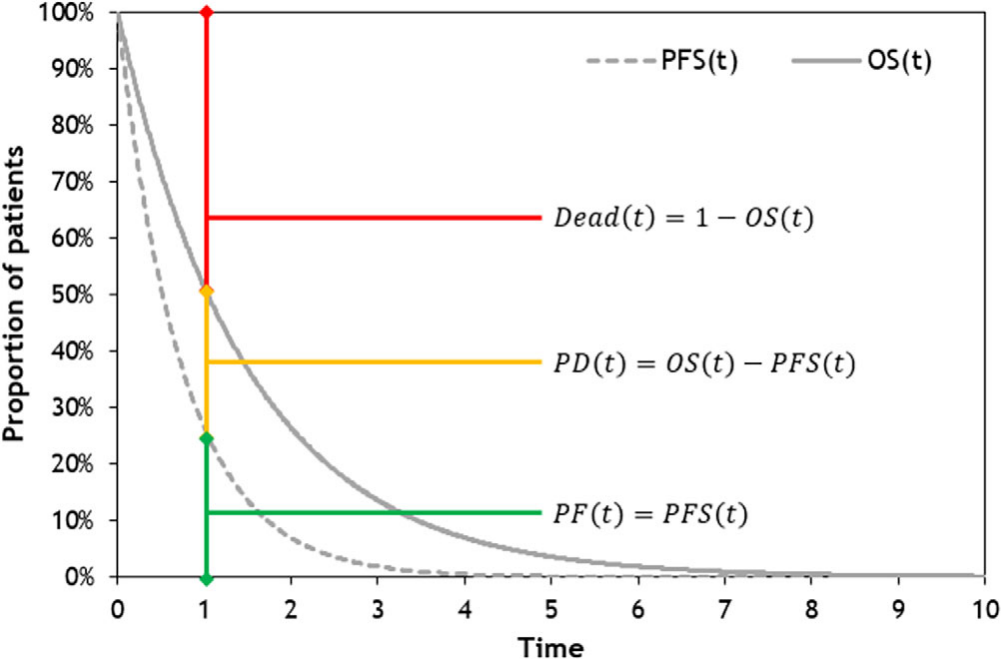
Model structure. A partitioned‐survival model was chosen based on the need to capture the most clinically important outcomes for mMCC patients—OS, PFS and ToT, which were obtained from the JAVELIN Merkel 200.^11^, ^12^, ^13^ This model allowed transition from three key mutually exclusive health states to survival: progression‐free disease, progressed disease, and death. In addition, a time‐to‐death approach was included in the model, which allowed variation between three time periods before death. mMCC, metastatic Merkel cell carcinoma; OS, overall survival; PD, progressed disease; PF, progression‐free; PFS, progression‐free survival; *t*, time; ToT, time on treatment

A de novo partitioned‐survival excel‐based model for mMCC was used for adaptation in Taiwan.^17^ This report was written based on the ISPOR Consolidated Health Economic Evaluation Reporting Standards^18^, ^19^ The conceptual structure considers three key mutually exclusive health states related to survival: progression‐free disease, progressed disease, and death (Figure 1). Transitions between model health states are informed by the area under progression‐free survival (PFS) and OS curves derived from the JAVELIN Merkel 200 data. The proportion of patients in the dead state is estimated by 1 minus the OS, the proportion with progressed disease is estimated by OS minus PFS, and the proportion with progression‐free disease is taken directly from PFS estimates in the clinical trial.

The model was adapted in Taiwan using a lifetime horizon of 40 years in order to ensure that all important costs and outcomes would be captured. This time horizon for estimating clinical and cost effectiveness is sufficiently long to reflect differences in costs and outcomes between the avelumab and the comparators.^20^ The choice of time horizon was accepted by the Taiwanese *Health Technology Assessment* and Taiwanese medical oncology experts consulted. These are practicing experts from Taipei Mackay Memorial Hospital, National Cheng Kung University Hospital, and Kaohsiung Chang Gung Memorial Hospital with extensive experience treating mMCC. Furthermore, the model used a cycle length of 1 week which is short enough to accurately reflect the timing of model costs and outcomes.^12^, ^13^, ^17^

The data of clinical efficacy, safety, and patient utilities were obtained from the JAVELIN Merkel 200 trial,^11^ literature review, and Taiwanese clinical expert opinion. Cost‐utility analysis was performed; and results were presented as cost per quality‐adjusted life year (QALY) gained, which is also known as the incremental cost‐effectiveness ratio (ICER). An annual discount rate of 3% for both costs and QALYs was aligned with the Taiwanese reference case in the base case model.

### 2.2 Comparator treatments

The model adaptation allowed to include both chemotherapy regimens (carboplatin etoposide, carboplatin paclitaxel, cisplatin etoposide, cisplatin paclitaxel, cyclophosphamide doxorubicin vincristine [CDV], doxorubicin, liposomal doxorubicin, paclitaxel, topotecan)^21^, ^22^ and best‐supportive care (BSC) as comparators. The main assumption made in the model was about equivalent efficacy of chemotherapy regimens and BSC since there are few data to compare efficacy between chemotherapy regimens, and no data for the outcomes associated with BSC^14^ Due to the rarity of mMCC, thorough validation of assumptions was made by three clinical experts who have treated mMCC cases in Taiwan. These experts are from Taipei Mackay Memorial Hospital, National Cheng Kung University Hospital, and Kaohsiung Chang Gung Memorial Hospital. Stringent quality‐control was checked by health economists throughout the study process. Hence, this ensured that outputs from the model were reflective of clinical expectation.

### 2.3 Avelumab treatment

Efficacy data informing the PFS and OS for patients receiving avelumab were obtained from the JAVELIN Merkel 200 trial.^11^, ^12^, ^13^ As JAVELIN Merkel 200 is a single‐arm trial, observational data sources were used to inform comparator outcomes. Other than conventional chemotherapy, there are no newly approved treatments for patients with mMCC in Taiwan. The JAVELIN Merkel 200 trial considers two parts: A (treatment‐experienced patients) and B (treatment‐naïve patients). For part A, all patients (n = 88) had been followed up for a minimum period of 36 months (data cut‐off date: September 2018). At the data cut‐off date of May 2019, the minimum followed‐up period was 15 months for all patients (n = 116) in part B.

### 2.4 Spline‐based survival models

Spline models with three functional forms: hazard, odds and normal; and with one, two or three intermediate knots were explored. Based on these candidate models for OS and PFS, the spline “1‐knot hazard” model was selected to inform the model base case for both outcomes for both cohorts. For OS, the simple parametric models failed to fully realize the long‐term survival estimate for PFS. The spline “1‐knot‐hazard” model provided one of the best statistical goodness of fit compared with other spline models, and exhibited plausible longer‐term extrapolations. For PFS, the same functional form was chosen owing to its superior visual goodness‐of‐fit compared with the other spline models, and for consistency with the approach used to model OS. The “1‐knot hazard” spline was also selected to inform time on treatment (ToT) for both cohorts because it could provide a good fit to the observed data, allow for consistency with OS and PFS, and show statistical goodness‐of‐fit scores that were comparable to the other potential models.

### 2.5 Adverse events (AEs)

The incidence of AEs among treatment‐experienced patients receiving avelumab was obtained from the JAVELIN Merkel 200 trial. For comparator regimens, the incidence rates of AEs were sourced from appropriate published literature^21^, ^22^, ^23^, ^24^, ^25^, ^26^, ^27^, ^28^, ^29^ and validated by clinicians. It was assumed that AE rates for treatment‐naïve and treatment‐experienced patients were the same. If AE data associated with chemotherapy regimens in mMCC patients were unavailable, evidence related to their use in the treatment of small cell lung cancer (SCLC) was used as the best proxy for likely AE rates due to similarities between the two diseases. If SCLC data were unavailable, melanoma data were used as a suitable alternative, as recommended by clinical experts. It was conservatively assumed that patients on BSC did not experience any AE.

### 2.6 Treatment costs

All costs are presented in New Taiwan dollars (NT$). US$1 was assumed equal to NT$30 by using the exchange rate extracted on February 13, 2020.

Avelumab is available as a 200 mg vial and is administered at a target dose of 10 mg/kg by a 1‐hour intravenous infusion once every 2 weeks until confirmed disease progression, unacceptable toxicity, or occurrence of any other criterion for withdrawal. The average weight for mMCC patients was assumed to be 60 kg.^30^ In the model, the cost per vial was set to US$1039.43, and the cost per mg was US$5.20. The relative dose intensity (RDI) of avelumab was applied as 95.43% as derived from patient‐level data from the JAVELIN Merkel 200 trial. The average dose for avelumab was calculated via the method of moments, and included vial wastage as 600 mg. The average cost per treatment with avelumab was US$3118.30.

The model applied the cost for chemotherapy regimens in accordance with the customized split of chemotherapy regimens used in Taiwan. A chemotherapy administration cost of US$53.30 per outpatient visit was applied. Patients receiving combination chemotherapy regimens were conservatively assumed to incur only one administration cost per visit. For avelumab, administration cost was incurred with every two‐week treatment, whereas for chemotherapy regimens, a weekly administration cost was applied. Chemotherapy can be given for a maximum of six treatment cycles, and therefore was applied to patients in the first 18 cycles of the model. The schedule of paclitaxel is once every 4 weeks, and is different from those of other chemotherapy drugs. Patients on BSC were assumed not to incur any antineoplastic drug costs as no drugs are used for BSC.

The maximum duration of treatment for each chemotherapy regimen was sourced from published literature.^21^, ^22^, ^23^, ^24^, ^25^, ^26^, ^27^, ^28^, ^29^ The first method used to model ToT was fitting parametric model of Kaplan‐Meier survival curve. The second method involved seeking clinical expert opinion to establish how avelumab would be expected to be administered in clinical practice, particularly in the long term.

### 2.7 Medical resource use and costs

The costs of monitoring and resource use were identified from Taiwanese specific sources such as National Health Insurance Administration Online,^31^ Nation Health Insurance Administration Medical Service Online,^32^ and National Health Insurance Annual Medical Expenses Reports^33^ (Table 1).

**TABLE 1:** Costs

Resource Use	Unit Costs (US$)	References
IV administration	53.30	NHIA Medical Service^32^
Outpatient visit	8.67	
CT scan	167.83	
Full blood count	6.67	
Liver function tests	6.33	
Renal function tests	2.67	
Thyroid function tests	10.00	
Radiotherapy	404.10	
End‐of‐life care costs		
Administration expenses	2538.53	Chang et al^34^
Outpatient care expenses	195.50
AE costs		
Anemia	99.27	NHIA Annual Medical Expense Reports^33^
Dyspnea	31.87	
Fatigue	31.87	
Febrile neutropenia	500.60	
Low hemoglobin	31.87	
Hyponatremia	72.30	
Infections	48.60	
Leukopenia	500.60	
Lymphopenia	500.60	
Muscle pain	28.43	
Nausea/vomiting	31.87	
Neutropenia	500.60	
Low platelets	500.60	
Sensory neuropathy	44.73	
Thrombocytopenia	500.60	
Hair loss (any grade)	20.67	

Abbreviations: AE, adverse event; CT, computerized tomography; IV, intravenous; NHIA, National Health Insurance Administration; US$, US dollar.

Data regarding the medical resource use frequencies for patients with mMCC are lacking due to the rarity of the disease. Therefore, estimates of resource use frequency were initially obtained via the literature review using SCLC as a suitable alternative as validated by Taiwan oncology experts.

Patients receiving BSC, or patients in the post‐progression health state were expected to incur the cost of one outpatient visit every 2 months. The resource use frequency for progression‐free patients receiving avelumab was modelled as every treatment cycle for outpatient visit, full blood count, liver function tests, renal function tests, and thyroid function tests. CT scan was modelled to be every 3 months. The resource use frequency was determined for progression‐free patients receiving chemotherapy using clinical validation and the only difference was that thyroid function tests were not included.

The average cost of end‐of‐life care for terminal cancer patients in the last 30 days was obtained from Taiwanese literature.^34^ The costs for end‐of‐life administration and outpatient care were US$2538.53 and US$195.50, respectively. Hence, the average cost for end‐of‐life care was considered as US$2734.06. Inflation of end‐of‐life care costs was addressed.^17^

### 2.8 Health‐related quality of life

HRQoL data were collected in the JAVELIN Merkel 200 study via the EQ‐5D‐5L questionnaire, and HRQoL was assessed at baseline, at 6‐weekly intervals during treatment period, and at the end‐of‐treatment visit. Utility scores were valued using the EQ‐5D‐5L value set for Korea.^35^ The Korean utility scores were also validated by Taiwanese clinicians. Values were linked to patients' response status to obtain utility values for progression‐free and post progression disease states. Endpoints were assessed by an independent endpoint review committee (IERC) and validated by Taiwanese medical oncology experts. The health state utility values for patients treated with comparators were assumed to be the same as those for patients treated with avelumab. Utility analysis was conducted using a time‐to‐death approach. Models were constructed to allow utility variation using up to three time periods before death: 34 or less days before death, 35‐265 days before death and more than 265 days. The results of regression analysis and the utility values applied in the model are given in Table 2.

**TABLE 2:** Utility by time‐to‐death: results of regression analysis and health state utility values assumed in the model

Health State/Coefficient	Estimate	*P* value
266+ days to death	0.8019	<0.001
35–265 days to death	−0.0933	<0.001
0‐34 days to death	−0.3608	<0.001
Treatment experienced	−0.0348	0.201

The QALY decrement for avelumab was 0.000004 per cycle, owing to its relatively mild toxicity profile, compared with a QALY decrement of 0.000784 per cycle for chemotherapy. As the time‐to‐death utilities did not differentiate between patients receiving active treatment and patients not receiving active treatment, AE‐related disutilities were incorporated within the “progression‐free disease and on treatment” and “progressed disease and on treatment” health states, and calculated as QALY decrements.

### 2.9 Analyses

The utilities according to time‐to‐death were investigated within scenario analysis. One‐way sensitivity analysis (OWSA) was conducted to assess the sensitivity of cost‐utility results to individual parameters associated with uncertainty in the model. The key areas of uncertainty pertaining to model settings were utility values, resource use, frequency of outpatient visit and CT scan, and outpatient cost for treatment‐experienced patients. Probabilistic sensitivity analysis (PSA) was undertaken to explore the joint uncertainty of all model parameters, and their associated impact on cost‐utility results. PSA was performed by running 1000 iterations.

## 3 RESULTS

### 3.1 Base‐case results for treatment‐naïve patients

The base‐case results are shown in Table 3. Treatment‐naïve mMCC patients receiving avelumab were estimated to experience 3.49 more life years (LYs), 2.16 additional QALYs, and an incremental cost of US$97 116.13 per patient compared with those receiving BSC. The ICER for avelumab vs BSC was estimated to be US$44 885.06 per QALY gained. Compared with chemotherapy, avelumab was associated with 3.49 LYs gained, 2.20 incremental QALYs, and an incremental cost of US$94 437.10 per patient. The ICER for avelumab vs chemotherapy was US$42 993.06 per QALY gained.

**TABLE 3:** Base‐case results for treatment‐naïve and treatment‐experienced mMCC patients

Treatment	Total Costs (US$)	Total QALYs	Total LYs	Incremental, Avelumab vs Comparator			ICER (US$ per QALY Gained)	
				Costs (US$)	QALYs	LYs		
Base‐case results for treatment‐naïve mMCC patients								
Avelumab	100 281.93	3.518	5.426				
Drug	87 030.26						
Administration	1745.50						
MRU	8844.63						
AE	252.13						
End of life	2409.41						
Chemotherapy	Total 5844.83	1.322	1.937	94 437.10	2.197	3.489	42 993.06
Drug	2992.22						
Administration	0.00						
MRU	0.00						
AE	224.58						
End of life	2628.03						
BSC	Total 3165.79	1.355	1.937	97 116.13	2.164	3.489	44 885.06
Drug	0.00						
Administration	313.18						
MRU	0.00						
AE	224.58						
End of life	2628.03						
Base‐case results for treatment‐experienced mMCC patients								
Avelumab	82 025.46	3.107	5.135					
Drug	74 783.65							
Administration	2276.59							
MRU	1775.04							
AE	755.78							
End of life	2434.40							
Chemotherapy	5594.08	0.229	0.414	76 431.40	2.878	4.722	26 557.43	
Drug	2383.73							
Administration	0.00							
MRU	0.00							
AE	476.28							
End of life	2734.07							
BSC	3892.18	0.239	0.414	78 133.26	2.868	4.722	27 243.06	
Drug	0.00							
Administration	681.84							
MRU	0.00							
AE	476.27							
End of life	2734.07							

Abbreviations: AE, adverse events; BSC, best supportive care; ICER, incremental cost‐effectiveness ratio; LYs, life‐years; mMCC, metastatic Merkel cell carcinoma; MRU, medical resource use; QALYs; quality‐adjusted life‐years; US$, US dollar.

### 3.2 Base‐case results for treatment‐experienced mMCC patients

Treating treatment‐experienced mMCC patients with avelumab was associated with 4.72 LYs gained, 2.87 incremental QALYs, and an incremental cost of US$78 133.26 per patient compared with BSC, which resulted in an ICER of US$27 243.06 per QALY gained (Table 3). Avelumab extended patient's life by 4.72 year vs chemotherapy, corresponding to a gain of 2.88 QALYs. The incremental cost associated with the use of avelumab vs chemotherapy was US$76 431.40 per patient, and therefore the ICER between these two regimens was US$26 557.43 per QALY gained.

### 3.3 Scenario analyses

Scenario analyses were conducted on key model settings and assumptions. Alternative assumptions around ToT contributed greatly to model uncertainty. Using alternative parameterizations for ToT caused the ICERs of avelumab vs chemotherapy to range between US$24 835.20 (exponential) and US$32 567.13 (log‐logistic). OS extrapolations produced variable results, with the most pessimistic, yet clinically plausible, extrapolation resulted in an ICER of US$38 483.10 for avelumab vs chemotherapy for the treatment‐experienced cohort of patients.

### 3.4 One‐way sensitivity analysis (OWSA)

**FIGURE 2:**
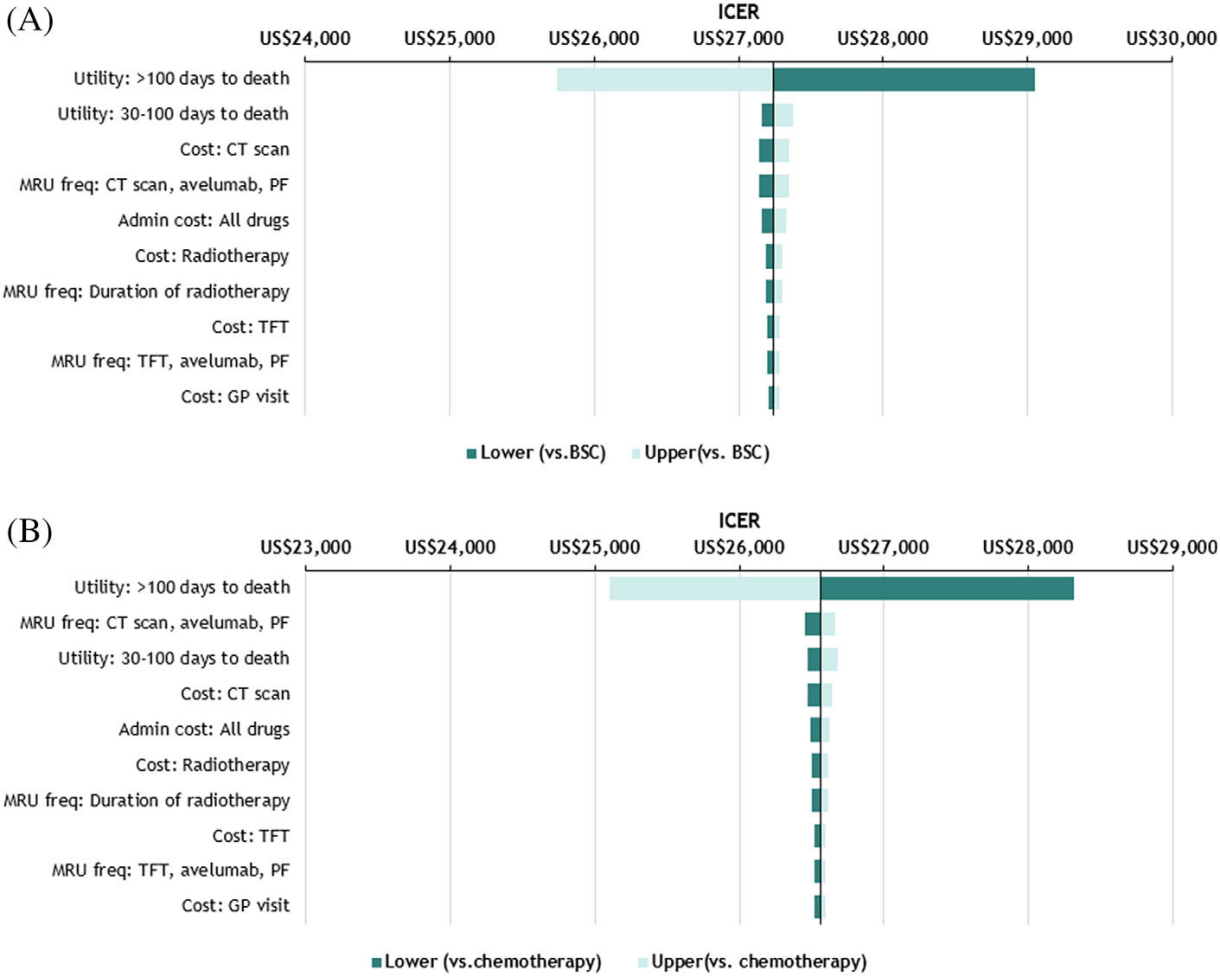
OWSA: (A) avelumab vs BSC for treatment experienced mMCC (in US dollar), and (B) avelumab vs chemotherapy for treatment‐naïve mMCC (in US dollar). BSC, best supportive care; CT, computed tomography; GP, general practitioner; ICER, incremental cost effectiveness ratio; MRU, medical resource use; OWSA, one‐way sensitivity analysis; PF, progression‐free; TFT, thyroid functional test; US$, US dollar

Figure 2 presents the results of OWSA for avelumab vs BSC in treatment‐experienced mMCC patients and avelumab vs chemotherapy in treatment‐naïve mMCC patients. Utility in >100 days to death was the most influential factor within the model, and other variables had a minor influence on the ICER.

### 3.5 Probabilistic sensitivity analysis (PSA)

**FIGURE 3:**
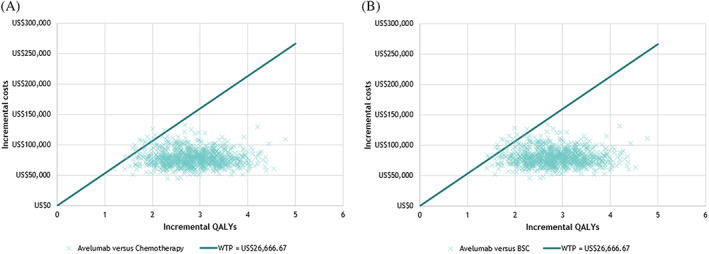
PSA scatterplot: (A) avelumab vs chemotherapy, and (B) avelumab vs BSC. BSC, best supportive care; QALY, quality‐adjusted life year; US$, US dollar; WTP, willingness‐to‐pay

For PSA, the model was run using 1000 iterations, at which point the model results were shown to be sufficiently stable for treatment‐experienced patients. The PSA illustrated that at a willingness‐to‐pay (WTP) threshold of US$53,333.33 per QALY gained, avelumab was associated with a 99% probability of being cost‐effective vs BSC (Figure 3A) and chemotherapy (Figure 3B). The spline “1‐knot hazard” model was applied to the OS, PFS, and ToT inputs of the PSA.

## 4 DISCUSSION

### 4.1 Importance of avelumab for the treatment of mMCC

Immune checkpoint inhibitors (ICIs), such as anti‐cytotoxic T lymphocyte antigen 4 (CTLA‐4) and anti‐programmed cell death 1 (PD‐1)/programmed cell death‐ligand 1 (PD‐L1) monoclonal antibodies, block the interactions between cancer cells and the immune system to enhance immune response to the tumor by rebalancing immune surveillance and immune evasion. Nowadays, ICIs have widely changed the standard of cancer treatment in various cancers.^36^

Based on the JAVELIN Merkel 200 trial, avelumab demonstrated significant benefit of response rate and survival outcomes for mMCC patients^11^, ^12^, ^13^ and provides an alternative treatment option for such life‐threatening disease.

### 4.2 Key findings and value of this study

The de novo economic analysis presented in this study detailed the cost‐utility of avelumab for both treatment‐experienced and treatment‐naïve mMCC patients. For both treatment‐naïve patients and treatment‐experienced patients, OWSA demonstrated no parameter values leading to increase the ICERs of avelumab vs BCS/chemotherapy beyond US$53,333.33 per QALY gained, which is 2 times the gross domestic product (GDP) per capita defined by World Health Organization (WHO) guidelines and local expert opinion (WHO. Cost effectiveness and strategic planning [WHO‐CHOICE].). The PSA scatterplot (Figure 3) demonstrated the spread of results, and the corresponding cost‐effectiveness acceptability curve (CEAC) illustrated that avelumab was associated with a 99% probability of being cost‐effective vs chemotherapy or BSC. The results of PSA are consistent with those presented in other countries.^17^, ^37^

To our knowledge, this is the first cost‐utility analysis of avelumab vs traditional treatments for mMCC in Taiwan. This analysis is unique because there are limited cost‐utility analyses of treatments for such rare cancer. There are two key limitations to this analysis. The first limitation relates to the rarity of mMCC in Taiwan. Such low disease prevalence may result in heterogeneity of disease diagnosis and treatment effect. The comparisons between Bavencio and BSC or chemotherapies were therefore limited due to the paucity of clinical data and lack of standard of care in mMCC. Adjusted comparisons were attempted to address the small sample sizes.^17^ The second limitation relates to the adoption of results from a single‐arm JAVELIN Merkel 200 trial design. It is difficult to interpret the response without a frame of reference for comparison. There may be an inability to distinguish between the effect of avelumab, a placebo effect, and the effect of natural history. Despite these limitations, single arm trial is commonly implemented in oncology for rare cancers evaluating treatments for which controlled trials are limited and placebos are deemed unethical.^38^ Uncertainties in long‐term survival outcomes for patients treated with avelumab will reduce as long‐term data from JAVELIN Merkel 200 becomes available. Taiwanese clinical expert validation was undertaken to mitigate this area of uncertainty within the model.

### 4.3 Cost‐effectiveness analyses of avelumab for mMCC in other countries

Avelumab also demonstrated cost‐effectiveness in the United Kingdom^17^ and Russia.^37^ At a WTP threshold of £50 000 per QALY gained for end‐of‐life treatments in the United Kingdom,^39^ avelumab was shown to be a cost‐effective treatment option compared with chemotherapy or BSC in treatment‐experienced and treatment‐naïve mMCC patients.^17^ The base‐case ICERs were £35 274 for the treatment‐experienced population, and £39 178 for the treatment‐naïve population.^17^ In Russia, avelumab was estimated to be cost effective vs chemotherapy in treatment‐experienced mMCC patients.^37^ This evidence is being assessed for the inclusion of avelumab in the Vital and Essential Drug List (VEDL) in Russia.^37^

## 5 CONCLUSION

This study demonstrated a de novo partitioned‐survival economic model confirming avelumab as a cost‐effective management option for treatment‐experienced and treatment‐naïve mMCC patients in Taiwan. For the analysis of both cohorts, the cost‐effectiveness results lie below the acceptable threshold of US$53,333.33 per QALY gained. This analysis was used as an evidence base for the national payer when considering reimbursement of avelumab for the treatment of mMCC patients in Taiwan.

## CONFLICT OF INTEREST

This research was financially supported by Merck Ltd., Taipei, Taiwan; an affiliate of Merck KGaA, Darmstadt, Germany, as part of an alliance between Merck KGaA and Pfizer. Amy Y. Lin and Connie Goh are employees of Merck Ltd., Taipei, Taiwan; an affiliate of Merck KGaA, Darmstadt, Germany. Anne Chang was an employee of Merck Ltd., Taipei, Taiwan at the time the analysis was conducted, Roberto Palencia is an employee of Merck KGaA, Darmstadt, Germany.

## AUTHOR CONTRIBUTIONS

**John W.C. Chang:** Conceptualization; formal analysis; investigation; resources; supervision; writing‐original draft; writing‐review & editing. **Jason Hsu:** Data curation; formal analysis; methodology; software; validation; visualization; writing‐original draft; writing‐review & editing. **Chiao‐En Wu:** Conceptualization; formal analysis; methodology; validation; visualization; writing‐original draft; writing‐review & editing. **Connie Goh:** Formal analysis; funding acquisition; methodology; project administration; resources; validation; writing‐original draft; writing‐review & editing. **Patrick Chou:** Data curation; formal analysis; investigation; methodology; project administration; software; validation; visualization. **Kaitlin Kuo:** Conceptualization; data curation; formal analysis; methodology; project administration; resources; software; validation; visualization; writing‐review & editing. **Anne Chang:** Formal analysis; methodology; resources; validation; writing‐review & editing. **Roberto Palencia:** Conceptualization; data curation; formal analysis; funding acquisition; investigation; methodology; project administration; resources; software; supervision; writing‐original draft; writing‐review & editing.

## ETHICAL STATEMENT

Institutional ethics approval and patient consent were not required for this study.

## Data Availability

The data that support the findings of this study are available from the corresponding author upon reasonable request.
